# Assessing the Potential of Lateritic Clayey Soils for Road Infrastructure in Tropical Regions

**DOI:** 10.3390/ma18081804

**Published:** 2025-04-15

**Authors:** Antônio Carlos Rodrigues Guimarães, Albeds Mesquita Povuação, Gabriel de Carvalho Nascimento, Sergio Neves Monteiro, Lisley Madeira Coelho

**Affiliations:** 1 Department of Fortification and Construction Engineering, Military Institute of Engineering-IME, Praça General Tibúrcio, 80, Urca, Rio de Janeiro 22290-270, Brazil; guimaraes@ime.eb.br (A.C.R.G.); mesquita.albeds@ime.eb.br (A.M.P.); 2 Department of Agricultural Engineering and Environment, Fluminense Federal University, Rio de Janeiro 24210-240, Brazil; gabrielcn@id.uff.br; 3 Department of Materials Science, Military Institute of Engineering-IME, Praça General Tibúrcio, 80, Urca, Rio de Janeiro 22290-270, Brazil; snevesmonteiro@gmail.com

**Keywords:** permanent deformation, resilient modulus, lateritic fine soil

## Abstract

Lateritic soils, characterized by complex mineralogy, a high degree of weathering, and a distinctive structure, are widely distributed in tropical regions. However, their use in pavement layers is often restricted due to conservative soil classification methods that may not fully represent their mechanical potential. This study evaluates the geotechnical behavior of a lateritic clay from a small town in São Paulo, referred to in this article as Purple Clay, with a focus on its permanent deformation (PD) and resilient modulus (RM). Repeated load triaxial tests, along with X-Ray Diffraction (XRD) and Scanning Electron Microscopy (SEM), were conducted to assess the soil’s mechanical response and microscopic structure. The results indicated that the high concentration of iron oxides contributed to increased cohesion and mechanical strength. When compacted at intermediate Proctor energy, the Purple Clay exhibited RM values comparable to some granular materials reported in the literature, highlighting its potential for pavement applications. However, under higher stress levels, PD was up to 42% greater than that of reference materials, emphasizing the influence of loading conditions on its behavior.

## 1. Introduction

Tropical and subtropical zone soils exhibit distinct mechanical parameters and behaviors compared to those found in temperate regions, primarily due to climatic conditions that intensify the weathering process. Among these soils, lateritic soils stand out due to their complex mineralogy, high degree of laterization—resulting from secondary physical–chemical weathering processes—and unique structural characteristics. These soils are widely distributed in regions with hot and humid climates [1,2,3,4]. The mineralogy of lateritic soils is characterized by the predominance of iron and aluminum oxides, as well as secondary minerals such as kaolinite, gibbsite, and hematite [5]. Depending on their degree of consolidation, these materials form distinct horizons along the soil profile, ranging from the saprolitic layer—which retains structural traces of the parent rock [6]—to iron-rich horizons that are often covered by a fine clay layer [7,8].

The predominant occurrence of lateritic soils is within the tropical belt, between the Tropics of Cancer and Capricorn, extending into adjacent areas such as the extreme western portion of South America, southwestern Africa, the desert regions of north-central Africa, the Arabian Peninsula, and the interior of Australia [9]. In these regions, lateritic soils are abundant and low-cost materials, with variations depending on climate, parent rock, topography, and formation time.

Despite their widespread occurrence, the characterization of lateritic soils still relies on traditional classification systems such as the California Bearing Ratio (CBR), the Unified Soil Classification System (USCS), and the Highway Research Board (HRB) classification [10,11,12,13,14,15,16,17,18]. However, these methods often fail to capture the specific geotechnical behavior of lateritic soils, particularly fine-grained ones, which are frequently disregarded for use in base and sub-base layers of road pavements due to their high plasticity index (PI) and low CBR values, preventing them from meeting regulatory requirements [19].

This limitation is evident in studies such as Kamtchueng et al. [20], which show that lateritic soils in Cameroon are often classified within AASHTO groups A-7-5 and A-2-7, indicating poor to marginal quality for pavement applications. Likewise, Olofinyo et al. [12] reported significant variability in the geotechnical properties of lateritic soils in Nigeria, where clay-rich samples were categorized as unsuitable for base layers despite their potential for improvement through stabilization. These cases highlight how traditional classification criteria impose restrictive thresholds that do not necessarily reflect the in situ performance of these materials under traffic loading.

Additionally, the reliance on empirical indices such as the CBR overlooks critical aspects of mechanical behavior under real traffic conditions. Studies by Onana et al. [11], Mengue et al. [14], and Foko Tamba et al. [16], for example, focus on strength and compaction but neglect stress-dependent responses. Moreover, the lack of permanent deformation assessments limits the understanding of long-term performance. Nevertheless, evidence suggests that lateritic soils, when properly treated or compacted under controlled conditions, can achieve mechanical properties suitable for base layers, challenging the restrictive criteria of conventional classifications [21]. Given these shortcomings, incorporating advanced laboratory methodologies, such as triaxial cyclic loading tests, is crucial for a more realistic evaluation of lateritic soils. This methodological shift can optimize their use in pavement design, overcoming the overly conservative classification frameworks that currently restrict their application.

In addition to the need for advanced mechanical testing, the classification of lateritic soils faces challenges related to their unique particle aggregation and grain size distribution. Nogami and Villibor [5] emphasize that the strong aggregation of clay particles complicates the accurate determination of grain size distribution, rendering traditional methods poorly representative of the actual behavior of lateritic soils. Moreover, these classification systems often group materials with distinct geotechnical behaviors into the same categories, reinforcing the need for more specific approaches.

To address this gap, Nogami and Villibor [22] developed the Mini Compacted Tropical (MCT) method, which enables a more accurate assessment of the mechanical and hydraulic properties of these soils. By employing tests that replicate in situ conditions, the MCT method overcomes the limitations of traditional classification systems and has already been adopted by the Brazilian National Department of Transport Infrastructure (DNIT) under standard DNIT 259 [23]. This facilitates the proper identification and utilization of soils that would otherwise be discarded, contributing to the sustainability and economic feasibility of road construction projects.

The inadequacy of conventional assessment methods can lead to uncertainties regarding the strength of lateritic soils in the field. However, several studies indicate that these materials can exhibit satisfactory behavior when used in pavement structural layers, either in their natural state or combined with aggregates [24,25,26], and even applied in pavement layers without stabilization, particularly on roads with low to medium traffic volumes [27,28].

A practical example of this is the state of São Paulo, Brazil, whose road network spans approximately 200,000 kilometers, of which only 32,000 are paved [29]. Over the past five decades, more than 20,000 kilometers of road bases have been constructed using lateritic soils, demonstrating their feasibility [30]. In addition to road applications, studies indicate that lateritic soils can be used in railway infrastructure, such as sub-ballast layers, due to their favorable mechanical properties [31]. However, the proper characterization of these soils still requires further investigation to consolidate their scientific foundation and optimize their use.

In this context, repeated load triaxial (RLT) tests emerge as a fundamental tool for more realistically assessing the mechanical response of these soils under cyclic loading, enabling a more precise and representative characterization of their mechanical behavior [21,32,33,34,35,36]. Research indicates that lateritic soils can exhibit favorable mechanical behavior, with low levels of permanent deformation (PD) and high values of resilient modulus (RM) [21,37,38,39].

Studies have shown that, despite a low CBR value, certain Brazilian lateritic soils exhibit excellent behaviour in terms of RM and PD, making them suitable for use in pavement base layers [27,40]. Additionally, field tests conducted by Guimarães et al. [41] corroborated the predicted mechanical properties for a sandy lateritic soil, showing its low penetration rate and reduced PD. In this sense, Almeida et al. [26] observed that the incorporation of sand did not yield significant benefits for clayey lateritic soils, reinforcing the importance of carefully evaluating the mechanical behavior of these materials. The studies by Silva et al. [38] and Freitas et al. [39] confirm the high performance of lateritic soils in RM and PD, highlighting their resilience even under varying moisture conditions. The stabilization of PD was observed in all conditions, reinforcing the viability of these soils for pavement applications.

Given this context, this study investigates the geotechnical properties of a lateritic clay from the São Paulo region, Brazil, focusing on its physical–chemical, granulometric, and morphological characteristics. To evaluate its mechanical behavior, compacted specimens were subjected to permanent deformation (PD) and resilient modulus (RM) tests under dynamic triaxial loading. Additionally, X-Ray Diffraction (XRD) and Scanning Electron Microscopy (SEM) analyses were performed to better understand the interaction between soil constituents. The results are compared with the existing literature to assess the feasibility of using this material in road pavements. Moreover, the mechanical behavior of Purple Clay is analyzed through the shakedown theory, classifying its response under cyclic loading. This approach enhances the understanding of its resistance to permanent deformation, reinforcing its potential application in pavement layers.

## 2. Materials and Methods

### 2.1. Materials

**Figure 1:**
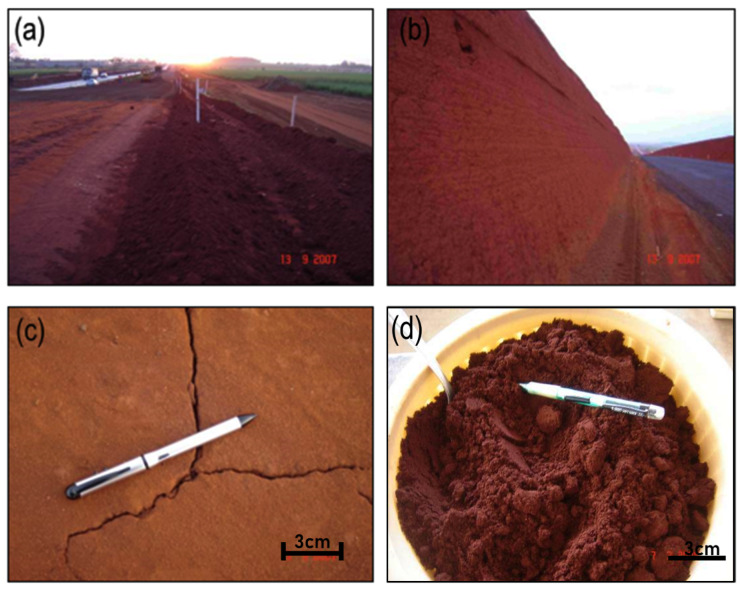
(**a**) General view of the embankment and sub-base of the pavement on the highway studied. (**b**) Aspect of a cutting slope made up of Purple Clay. (**c**) Cracking typical of fine lateritic soils observed in the Purple Clay sub-base layer. (**d**) Sample of Purple Clay at optimum humidity.

The material analyzed in this study is a lateritic soil typical of the Ribeirão Preto region in the state of São Paulo, Brazil, referred to in this article as Purple Clay. The sample was collected from the natural subgrade of the SP-333 highway, along the stretch between Ribeirão Preto and Serrana, specifically at km 38.9. At this location, the material was used in the sub-base layer of the pavement, as illustrated in Figure 1.

To ensure a representative sample for laboratory testing, approximately 200 kg of soil was collected from a cut slope at a depth of 3 m, corresponding to the deep B horizon—a characteristic layer of tropical soils. This depth was selected to obtain a material with a more stable mineralogical composition, less influenced by organic matter and surface weathering.

**Figure 2:**
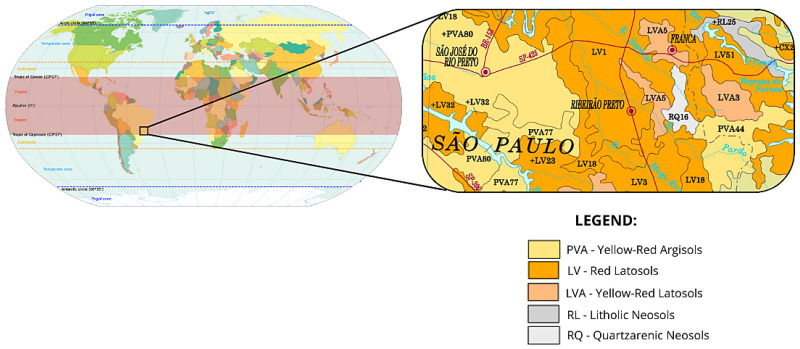
World map indicating tropical and subtropical regions, highlighting the Ribeirão Preto-SP region and the distribution of predominant soils in the area. Adapted from KVDP (2013) [42] and Embrapa (2001) [43].

Based on the analysis of the pedological map (see Figure 2), the sample belongs to the Red Latosol family, which predominates in the region where the SP-333 highway runs. The geological map also indicates the presence of extrusive magmatic rocks from the Serra Geral Formation, formed by fissural volcanism. These rocks consist of tholeiitic basalts and diabase dykes, interspersed with layers of aeolian sandstone (Arenito de Botucatu). The influence of these geological formations gives the soil its distinctive purplish-red color, which explains its common designation as Purple Clay.

In the modern Brazilian pedological classification, Latosols are subdivided into four suborders: Red Latosol, Red-Yellow Latosol, Yellow Latosol, and Bruno Latosol. The Red Latosol suborder includes soil classes formerly known as Purple Latosol and Dark Red Latosol [44]. Among these, Eutroferric Red Latosols are widely recognized as “purple earths”. Unlike most Latosols, which typically have low natural fertility, these soils are nutrient-rich, making them an exception within this group.

In international classification systems, such as the U.S. Soil Taxonomy, these soils are generally classified as Oxisols [45]. This classification underscores their nature as highly weathered materials, characteristic of tropical regions.

### 2.2. Methods

#### 2.2.1. Sample Preparation

In the laboratory, the material was initially air-dried and then oven-dried at 80 °C to remove excess moisture, a temperature considered safe for preserving the soil’s mineral composition [46,47,48]. After drying, the soil was crumbled and sieved to ensure uniform granulometry.

Portions of approximately 4000 g were prepared and moistened to the optimum moisture content (OMC), determined through Proctor tests. The samples were then stored in sealed plastic bags to prevent moisture loss or variation.

To ensure proper homogenization, the soil was manually mixed in a 0.5 m^3^ container before being redistributed into the plastic bags. The material was kept in a humidity chamber until testing, maintaining consistent moisture levels and ensuring reliable results.

#### 2.2.2. Physical, Chemical, and Mineralogical Characterization

A fraction of the sample was used for particle size analysis in accordance with the DNER-ME 083 [49] and DNER-ME 080 [50] standards. The optimum moisture content (OMC) and maximum dry apparent specific mass ($\rho_{\mathrm{dmax}}$) were determined through compaction tests using normal Proctor energy and a tripartite mold [51]. Additionally, characterization tests were performed following the MCT methodology [22].

Chemical analysis included pH determination in water and 1 M KCl, as well as loss on ignition to quantify hydrated minerals and organic matter. The chemical composition was determined via spectrometry, measuring the concentrations of iron (Fe_2_O_3_), aluminum (Al_2_O_3_), silica (${\mathrm{SiO}}_{2}$), titanium (${\mathrm{TiO}}_{2}$), and potassium ($K_{2}O$) oxides, along with the insoluble residue. Weathering indices were calculated based on the Fe_2_O_3_ and ${\mathrm{SiO}}_{2}$ contents to characterize the lateritic nature of the soil.

Morphological analysis was performed using a Quanta FEG 250 scanning electron microscope BY FEI ((Brno, Czech Republic), with gold coating applied in a Leica ACE600 (Leica Microsystems GmbH, Wetzlar, Germany) vacuum chamber. Both belong to the electron microscopy laboratory (LME) of the Military Institute of Engineering (IME), Rio de Janeiro, Brazil. Images were acquired with a 20 kV electron beam, a working distance of 10.5–13 mm, a spot size of 5, and magnifications of 280× and 3000×, using a secondary electron detector.

For XRD analysis, pure laterite soil, laterite soil with sand, and sand samples were prepared on a monocrystalline silicon substrate. The test was conducted using the Xpert Pro MRD System (PANalytical B.V., Almelo, The Netherlands) with Co Kα radiation (1.789 Å), scanning from 20° to 55° at a speed of 4°/min, with an operating power of 40 mA × 40 kV. The tests were conducted in line focus configuration, using the X’Pert Data Collector software, version 2.2j (2010), to input the equipment’s operating parameters. The diffraction data was processed using the X’Pert HighScore Plus software, version 2.0a (2004).

#### 2.2.3. Mechanical Analysis

On the scheduled date for mechanical testing, a portion of the sample was removed from the wet chamber and immediately transferred for mechanical compaction in a tripartite mold (10 cm in diameter and 20 cm in height). The compaction energy was adjusted according to the specifications of each sample, varying between normal and modified levels, as required by the study and based on the DNIT 443 [51] standard.

**Figure 3:**
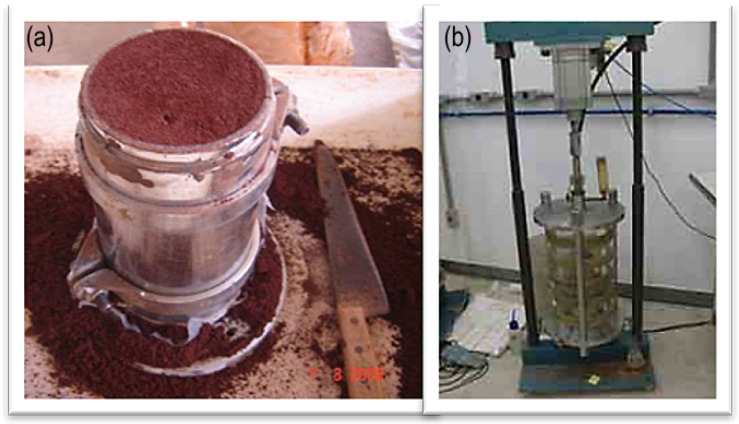
(**a**) Molded Purple Clay specimen and (**b**) specimen inside the triaxial apparatus.

The mechanical analysis included tests to determine key soil properties, such as the resilient modulus and permanent deformation. To simulate the material’s behavior under traffic conditions, repeated load triaxial (RLT) tests were performed in accordance with DNIT 179 [52] and DNIT 134 [53]. These standards establish procedures for evaluating the mechanical properties of soils, which are essential for predicting the performance of pavement materials. Figure 3 illustrates the molded Purple Clay specimen, while Figure 3b shows the specimen inside the triaxial apparatus.

The resilient modulus (RM) test was conducted following DNIT 134 [53], using a SIGEO dynamic triaxial test rig. The compaction energy was adjusted to correspond to the normal and intermediate Proctor compaction levels, in accordance with DNIT 443 [51].

During the RM test, eighteen pairs of confining stresses ($\sigma_{3}$) and deviatoric stresses ($\sigma_{d}$) were applied after the specimen conditioning phase. Each loading cycle lasted 1 second, with a 0.1 s load application period, at a frequency of 1 Hz. The specimens underwent three sets of conditioning stresses, each with 500 load cycles, followed by 18 additional stress pairs, with 100 load cycles per pair, totaling 3300 load cycles per test.

Permanent deformation (PD) tests were conducted on compacted specimens using the same energy as the normal Proctor test, under varying stress conditions detailed in Table 1. The loading frequency was 1 Hz, and the tests were performed with more than 150,000 load cycles, following the stress levels defined by DNIT 179 [54], which simulate typical traffic conditions equivalent to the standard road axle load of 8.2 tf.

It is worth noting that during specimen preparation, the material exhibited a strong tendency to adhere to the metal surfaces of the tripartite cylindrical mold, significantly complicating the demolding process. Figure 3a displays the molded Purple Clay specimen, while Figure 3b shows the specimen inside the triaxial apparatus.

It is worth noting that the progressive accumulation of stresses due to permanent deformation can lead to premature pavement failure, characterized by excessive rutting. This phenomenon has been widely studied to mitigate the accumulation of permanent deformation in pavement subgrades [55].

In this context, one of the analyses performed was the determination of the support limit of Purple Clay based on shakedown theory [21,56,57], which describes the behavior of materials under cyclic or repeated loading.

Shakedown theory suggests the existence of a critical stress threshold that separates stable from unstable conditions, playing a key role in predicting material performance under repeated loading. Based on this theory, material behavior can be classified into the following categories:

(A) Shakedown or Plastic Settling: The material undergoes plastic deformation over a finite number of load cycles. After this initial compaction phase, it responds in a fully elastic manner. (B) Intermediate Response: The material experiences high deformation during the initial load applications, but this deformation gradually decreases and stabilizes, resulting in a more uniform deformation rate. (C) Collapse: The material undergoes continuous plastic deformation with no tendency to stabilize. (AB) Intermediate Behavior: The sample exhibits significant initial deformations, followed by plastic accommodation. This behavior is similar to that observed in Brazilian fine soils, as reported by Guimarães and Motta [21].

## 3. Results and Discussion

### 3.1. Material Characterization

In terms of physical properties, Purple Clay consisted predominantly of clay (37%), followed by silt (25%) and sand (38%), with the sand fraction further divided into fine sand (29%), medium sand (8%), and coarse sand (1%), as shown in Table 2. The absence of a stony fraction characterizes the material as predominantly fine-textured, with a strong presence of clay particles.

Purple Clay exhibited an optimum moisture content (OMC) of 24.0% and a maximum dry density (γs) of 1.665 g/cm^3^, according to the normal Proctor test. The material had a liquid limit (LL) of 43.7%, a plastic limit (PL) of 16.6%, and a plasticity index (PI) of 27.1. Based on the MCT classification, it was identified as type LG’, with e’ = 1.08 and c’ = 1.81, indicating lateritic behavior. Conventional classification systems, such as USCS and HRB, do not adequately capture the specific characteristics of fine tropical soils. Under the HRB system, this material would be classified as A-7-6, while the USCS categorizes it as CH, both classifications typically associated with low-strength materials, often considered unsuitable for pavement layers [58]. However, the MCT classification suggests that this soil exhibits distinct mechanical properties that may justify its application in road engineering [59].

With regard to chemical characterization, Table 3 presents the results for Purple Clay. The measured pH values were 5.52 in water and 5.71 in 1 M KCl, indicating a slightly acidic environment, which is typical of lateritic soils subjected to intense weathering in humid tropical regions. The loss on ignition (8.39%) suggests the presence of hydrated minerals and organic matter.

The chemical composition indicates a soil enriched in iron and aluminum oxides, with Fe_2_O_3_ (25.5%) and Al_2_O_3_ (20.1%), while the silica content (${\mathrm{SiO}}_{2}$) was relatively low (13.0%), reflecting the progressive removal of silica, a characteristic process of laterization. The low concentrations of ${\mathrm{TiO}}_{2}$ (4.2%) and $K_{2}O$ (0.02%) further reinforce the intense leaching of more soluble constituents. The insoluble residue content of 24.6% suggests the presence of fractions resistant to chemical weathering. Finally, the calculated weathering indices ($K_{i}=1.10$ and $K_{r}=0.61$) confirm the lateritic nature of the soil, indicating an advanced degree of alteration and significant silica depletion.

**Figure 4:**
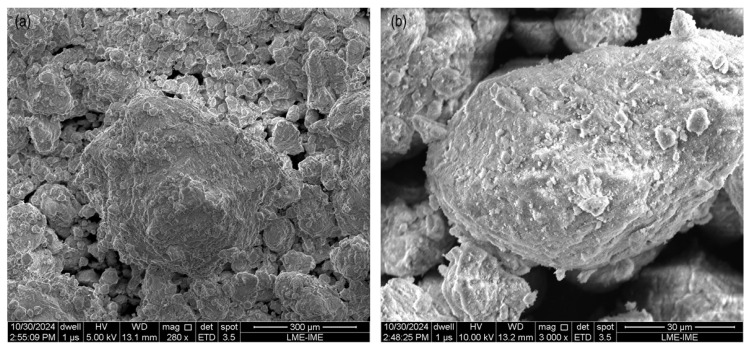
Micrographs of the Purple Clay sample at different magnifications: (**a**) 280× magnification; (**b**) 3000× magnification.

In addition to chemical characterization, the morphology of Purple Clay was analyzed using SEM. The images, shown in Figure 4a,b, reveal the highly porous and irregular structure of the soil. At 280× magnification, a typical “popcorn” or “sponge” arrangement is observed, characteristic of lateritic soils, as described by Villibor et al. [25]. This feature suggests the presence of agglomerates formed by iron and aluminum oxides, which influence the structural stability of the material. At 3000× magnification, the rough surface of the particles and the presence of interconnected voids become evident, potentially affecting both the permeability and mechanical behavior of Purple Clay.

**Figure 5:**
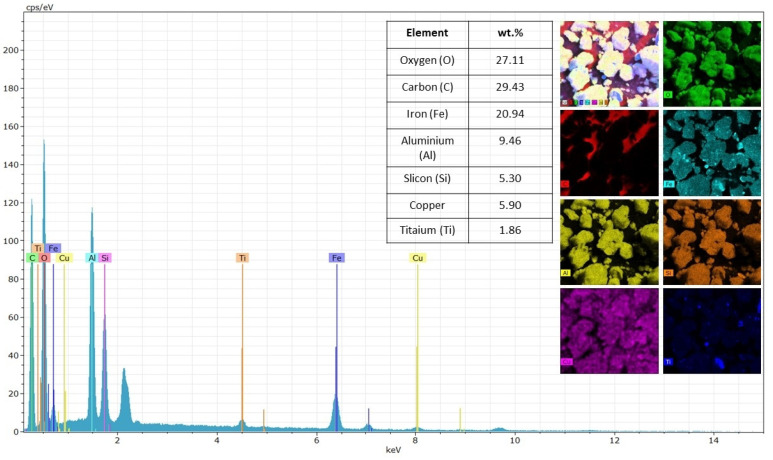
EDS spectrum of the Purple Clay sample.

Complementing the morphological analysis, Energy Dispersive X-ray Spectroscopy (EDS), illustrated in Figure 5, confirms the chemical composition of the sample, identifying elements such as oxygen (O), carbon (C), iron (Fe), aluminum (Al), silicon (Si), copper (Cu), and titanium (Ti). The predominance of oxygen, iron, and aluminum aligns with findings in the literature on lateritic soils, reinforcing the presence of iron and aluminum oxides as typical components of this material [5,19,60,61,62]. The high oxygen concentration suggests the occurrence of hydrated minerals, while the lower silicon content indicates the presence of secondary minerals. The presence of copper and titanium, albeit in smaller quantities, may be associated with the specific mineralogical composition of Purple Clay, as well as natural impurities or traces of secondary minerals.

**Figure 6:**
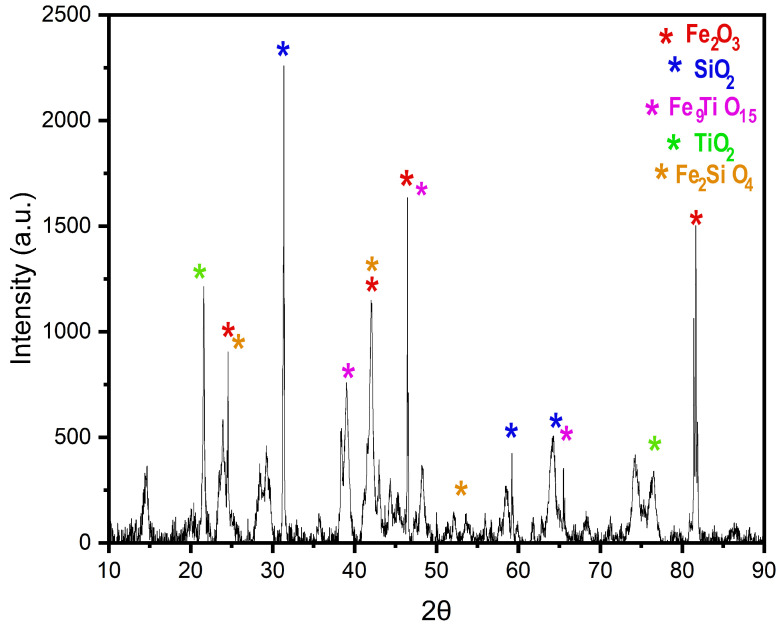
XRD Purple Clay.

XRD analysis of the studied sample (Figure 6) identified the presence of iron oxides (Fe_2_O_3_), quartz (SiO_2_), iron titanates (FeTiO_1_5), titanium dioxide (TiO_2_), and iron silicates (Fe_2_SiO_4_). These results align with previous studies on lateritic materials, indicating a matrix rich in metal oxides and silicates. Additionally, the mineral phases kaolinite, quartz, hematite, and goethite were identified, which are commonly found in lateritic soils [63]. Kaolinite, known for its non-expansive nature in the presence of water, enhances the material’s stability under humid conditions, in contrast to expansive clays that undergo significant volumetric changes, as discussed by Barbosa [64]. Similarly, Kamtchueng et al. [20], Santha Kumar et al. [65], and Santana et al. [66] also reported high levels of iron oxides and quartz in comparable samples, reinforcing the role of weathering in shaping the mineralogical composition.

The high-intensity peaks, particularly those associated with quartz and iron oxides, suggest that these compounds dominate the sample, directly influencing its mechanical and chemical properties. The strong presence of Fe_2_O_3_ aligns with the SEM morphological analysis, which revealed the formation of agglomerates and a highly porous structure, characteristic of lateritic soils. Additionally, the identification of FeTiO_15_ and TiO_2_ may indicate the occurrence of accessory titanium-bearing minerals, which were previously detected via EDS analysis.

The identified mineralogical distribution has significant implications for the material’s behavior in various geotechnical applications. The high concentration of iron oxides, such as goethite and hematite, tends to enhance soil cohesion and mechanical strength [25]. Furthermore, the presence of quartz may influence the material’s abrasiveness and durability, as highlighted by Benatti and Miguel [67], who noted that the cementation observed in lateritic soils results from the migration of particles and soluble chemical compounds in the unsaturated zone, leading to the formation of metastable structures with high porosity. Moreover, Guimaraes et al. [68] emphasized that the mineralogical characteristics of tropical soils contribute to their superior performance when used as pavement layers, particularly due to the partially irreversible cementation that occurs upon drying. Thus, the findings corroborate the existing literature and highlight the predominant mineralogical characteristics of these materials, which directly influence their mechanical behavior and stabilization potential.

### 3.2. Resilient Module (RM)

**Figure 7:**
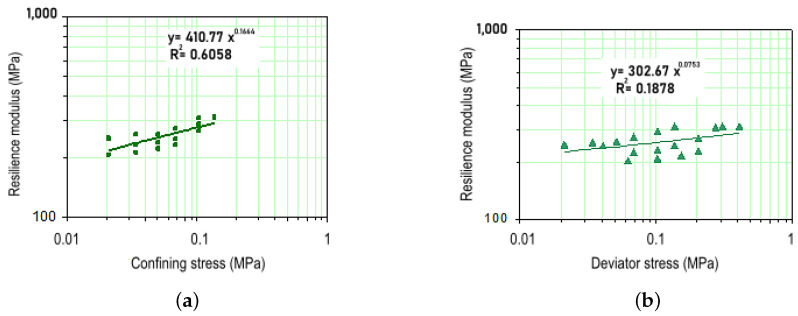
Resilient modulus (RM) variation: (**a**) with confining stress; (**b**) with deviator stress, for Purple Clay compacted using Standard Proctor energy.

The RM test results for specimens compacted with energy equivalent to the standard Proctor test are presented in Figure 7a,b, along with the corresponding equations. The average RM obtained was 258 MPa, a relatively high value for materials intended for subgrade or reinforcement layers.

Similar results were reported by Oguntayo et al. [69] in lateritic soils, where RM values ranged from 200.16 MPa to 233.50 MPa, indicating that these materials exhibit sufficient stiffness for subgrade applications.

The analysis of Figure 7a demonstrates that confining stress has a considerable influence on the material’s behavior, potentially increasing RM by up to 100% when varying from the minimum to the maximum values adopted in the tests [70]. A similar trend was observed by Samb et al. [71], who reported that the resilient modulus decreases with increasing deviatoric stress under constant confining pressure but increases when cement is added at similar moisture contents. These findings align with the results shown in Figure 7a, where confining stress significantly affects RM values, while deviatoric stress has little influence. This is further evidenced in Figure 7b, where the correlation coefficient obtained was extremely low.

**Figure 8:**
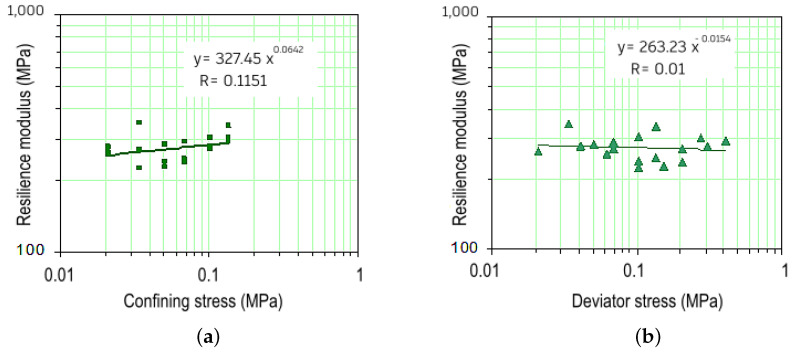
Resilient modulus (RM) variation: (**a**) with confining stress; (**b**) with deviator stress, for Purple Clay compacted using intermediate Proctor energy.

For the specimens compacted with energy equivalent to the intermediate Proctor test, the results are presented in Figure 8a,b. In this case, the average RM increased to 275 MPa. The correlation coefficients, close to zero, suggest that the resilient modulus remains practically constant, indicating minimal variation under different test conditions. Similar findings were reported by Samb et al. [71], who demonstrated that cement-stabilized lateritic soils exhibit enhanced mechanical properties, with RM values increasing significantly due to improved granular interaction. Additionally, studies have shown that chemical stabilization using cement or lime can further enhance soil stiffness.

**Figure 9:**
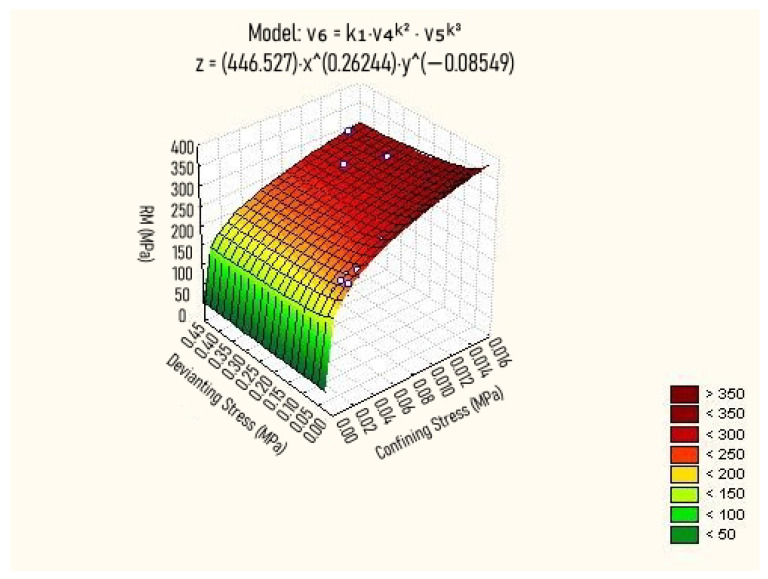
Variation in RM as a function of deviating and confining stress (Composite Model). Purple Clay.

The composite model, illustrated in Figure 9, evaluates the combined effects of deviatoric and confining stresses on the soil’s resilient modulus. For the lowest level of confining stress, RM reaches approximately 150 MPa. As confining stress increases, this value rises significantly, reaching around 350 MPa. This behavior indicates high stiffness for a fine-grained soil compacted with energy equivalent to the standard Proctor test. These results align with those obtained by Ki et al. [72], who developed predictive models for RM in lateritic soils from Burkina Faso and Senegal, highlighting the strong dependence of RM on confining stress.

Furthermore, the analysis of Figure 9 shows that deviatoric stress has a relatively small influence on RM variation. This behavior is described by Equations (1) and (2), which represent the composite model. The limited impact of deviatoric stress on RM was also noted by Mengue et al. [14], reinforcing the notion that confining stress is the primary controlling factor in the resilient response of compacted fine-grained soils.

(1) $$RM=2.971\cdot \sigma_{3}^{0.262}\cdot \sigma_{d}^{-0.085}\;\left({\mathrm{kgf}/\mathrm{cm}}^{2}\right)$$

(2) $$RM=446.5\cdot \sigma_{3}^{0.262}\cdot \sigma_{d}^{-0.086}\;\left(\mathrm{MPa}\right)\;R^{2}=0.855$$

As discussed by Moreira [73], tropical soils have demonstrated good mechanical performance in pavements, challenging conventional standards. Their findings indicate that, despite their fine-grained nature, the lateritic tropical soils of São Paulo exhibit favorable resilient behavior, making them suitable for pavement layers.

To contextualize the results of the combined model, which highlights the influence of confining and deviatoric stresses on the RM of Purple Clay, a comparison with previous studies is relevant. Neto et al. [74] determined RM values for base and subgrade layers of fine lateritic soils through back-analysis of existing pavements in São Paulo. Some of these results are presented in Table 4, alongside the RM values obtained in the present study through laboratory tests on Purple Clay.

As shown in Table 4, the RM of Purple Clay compacted with energy equivalent to the intermediate Proctor test aligns closely with the values reported by Neto et al. [74] for LA and LA’ type soils. Additionally, when compared to soils typically used in subgrade layers, Purple Clay exhibits a significantly higher RM, reinforcing its potential for pavement applications.

**Figure 10:**
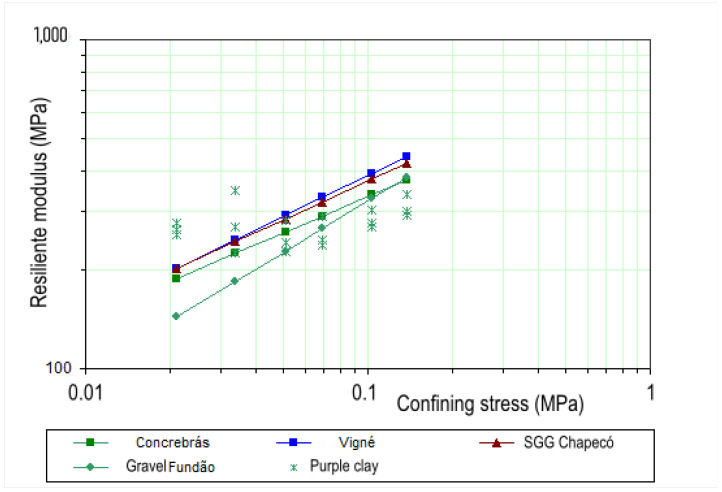
Variation in RM as a function of deviating stress. Purple Clay compacted with intermediate proctor energy.

The comparison of the RM values of Purple Clay with those of different types of graded crushed stone is presented in Figure 10. The data for Concrebrás and Vigné aggregates were obtained from Ramos and Motta [75], while the values for the graded crushed stone from Chapecó were sourced from Guimarães and Motta [76]. Additionally, the values for the Fundão aggregate come from recent studies conducted at the geotechnical laboratory of COPPE/UFRJ [21]. It is important to note that all graded crushed stones were compacted using energy equivalent to the modified Proctor test.

Given these results, it is relevant to consider an alternative commonly used to enhance the mechanical performance of lateritic soils: cement stabilization [77]. Caro et al. [78] demonstrated that the addition of cement to a sandy lateritic soil, classified as SL according to the SUCS system, resulted in a 63.22% increase in mechanical strength compared to the untreated material, highlighting the benefits of this technique in improving soil mechanical behavior.

Although this study does not evaluate the stabilization of Purple Clay with cement, it is worth emphasizing that its bearing capacity is already higher than typically expected for fine-grained soils, reinforcing its suitability for pavement applications. Additionally, Mengue et al. [14] demonstrated that incorporating cement into lateritic soils enables their use as base layers, increasing material stiffness and allowing for a reduction in the thickness of base and sub-base layers. Thus, cement stabilization, widely employed in studies involving lateritic soils, remains a well-established technique for enhancing their mechanical performance in road pavements.

Observing Figure 10, it is noted that the resilient modulus of Purple Clay is higher than that of crushed stones at the two lowest levels of confining stress (21 kPa and 34 kPa). However, as the confining stress increases, particularly at intermediate levels (51 kPa and 69 kPa), the RM values of the different materials become quite similar. At the highest levels of confining stress (103 kPa and 137 kPa), the RM of Purple Clay is lower than that of the graded crushed stones.

### 3.3. Permanent Deformation (PD)

**Figure 11:**
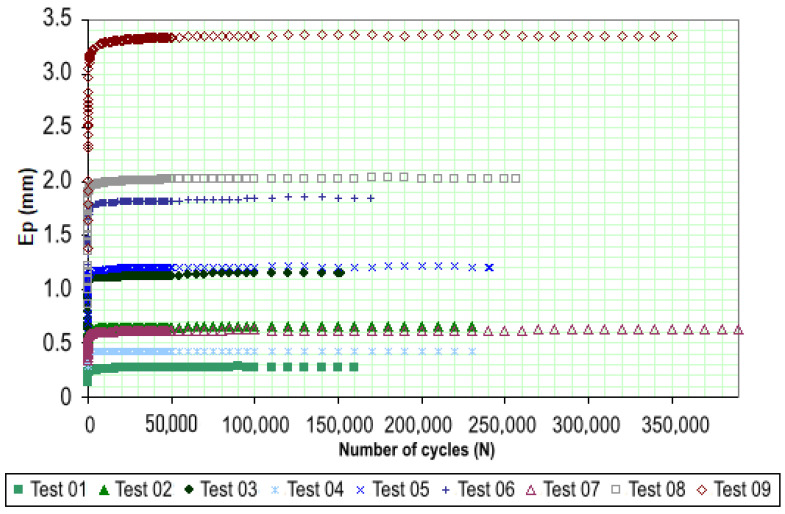
Total PD for Purple Clay.

In Figure 11, the results of the permanent deformation (PD) tests listed in Table 1 are presented, showing the total or accumulated PD of the 20 cm-high specimens as a function of the number N of load applications. Each test number in the figure corresponds to the conditions specified in Table 1, which include different stress levels ($\sigma_{d}$) and the stress ratio ($\sigma_{d}/\sigma_{3}$). For example, tests 1, 2, and 3 were conducted with stress levels of 40 kPa, 80 kPa, and 120 kPa, respectively, and with a constant stress ratio of ($\sigma_{d}/\sigma_{3}$) equal to 1. Similarly, tests 4, 5, and 6 were performed at higher stress levels, with each test identified by a specific combination of these parameters.

As shown in Figure 11, all specimens exhibited a clear trend of PD stabilization over the loading cycles, as indicated by the curves becoming nearly parallel to the horizontal axis. The primary difference in behavior among the tests is observed during the initial loading phase, up to approximately 5000 cycles, where the rate of PD accumulation increases with higher applied stress levels.

The maximum PD recorded was 3.4 mm in test 9, which was conducted with a deviatoric stress of 360 kPa and a confining stress of 120 kPa. This deformation represents the contribution of Purple Clay to the total rut depth in a pavement structure. Considering an allowable rut depth of 10 mm, this corresponds to a 34% contribution.

The stress states adopted in the tests allow for a more detailed analysis of the influence of deviator stress, particularly when the confining stress is kept constant. For instance, in tests 7, 8, and 9, which were conducted under the same confining stress ($\sigma_{3}=120$ kPa), the total permanent deformations were 3.4 mm, 2.0 mm, and 0.6 mm, corresponding to deviator stresses of 360 kPa, 240 kPa, and 120 kPa, respectively. This indicates that, when the confining stress remains constant, permanent deformation tends to increase as the $\sigma_{d}/\sigma_{3}$ ratio rises.

This behavior is consistent with the findings of Pascoal et al. [79], who also evaluated a lateritic clay compacted with standard energy and subjected to PD tests using a repeated load triaxial apparatus. However, under the same stress conditions ($\sigma_{3}=120$ kPa and $\sigma_{d}$ = 360 kPa), they reported a plastic deformation of 8.3 mm, whereas in the present study, the observed deformation was significantly lower, at 3.4 mm. Although both materials are classified as LG’ according to the MCT methodology and were compacted with the same energy level, this discrepancy suggests that even within the same geotechnical classification, intrinsic soil characteristics, such as particle size distribution, plasticity, and aggregate structure, can significantly influence mechanical behavior.

Furthermore, factors such as mineralogical variability and particle interactions may play a crucial role in the material’s response to repeated loads. This highlights the importance of expanding studies and datasets on lateritic soils to enable a more comprehensive characterization of their specific mechanical properties within a given classification.

A similar trend can be observed across the other stress stages. Another important aspect highlighted in Figure 11 is that in tests 1, 2, 3, 4, 5, and 7, the total permanent deformations remained below 1.0 mm. This suggests that the contribution of Purple Clay to potential pavement rutting would be minimal. These stress levels are fully consistent with the theoretical stresses expected for sub-base or subgrade layers in typical Brazilian pavements, even under wheel loads of approximately 18 tf.

The findings of this study can be compared with those of Almeida et al. [26], who investigated the PD behavior of pure laterite and its mixture with sand. Their results showed that pure laterite exhibited lower permanent deformation, even under higher stress levels, a behavior similar to that observed for Purple Clay in the present study.

**Figure 12:**
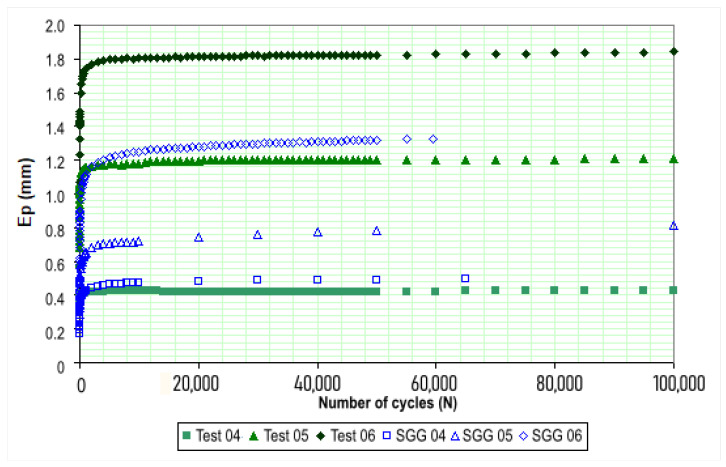
Comparison between the total PD exhibited by Purple Clay and graded crushed stones. Confining stress of 80 kPa.

Figure 12 presents a comparison between the total permanent deformation of Purple Clay and that of a graded aggregate from Chapecó, as studied by Guimarães and Motta [76]. The tests for both materials were conducted under similar conditions, with the numbering and corresponding stress states for the Chapecó aggregate following the same parameters established in Table 1.

An analysis of Figure 12 reveals that the permanent deformations of Purple Clay and the Chapecó graded aggregate are quite similar at lower stress levels. However, as the applied stress increases, Purple Clay exhibits a more pronounced rise in PD, with deformations up to 42% higher than those of the aggregate at the highest stress level.

### 3.4. Shakedown Analysis

The analysis of the shakedown phenomenon, performed using repeated load triaxial tests, served as a reference for the present study, drawing upon the works of Werkmeister et al. [57], Dawson and Wellner [56], and Werkmeister et al. [80].

**Figure 13:**
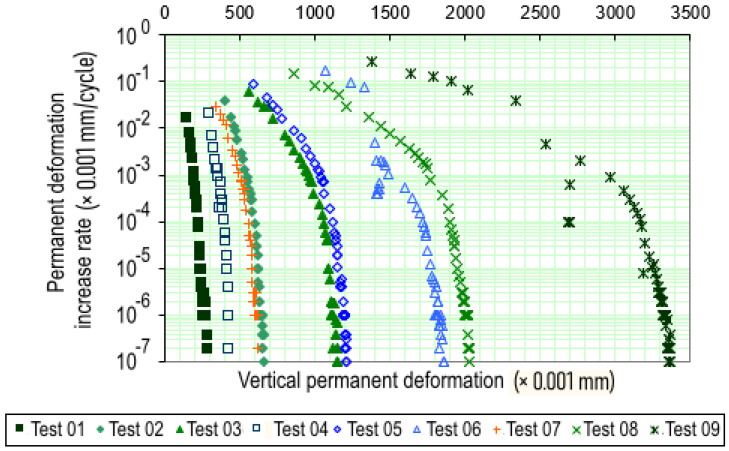
Shakedown occurrence investigation. Dawson and Wellner model [56].

In this approach, the rate of permanent deformation accumulation is evaluated as a function of the total vertical permanent deformation of the specimens, as illustrated in Figure 13.

Furthermore, this body of research provided the foundation for the British standard BS 13286-7:2004 Unbound and Hydraulically Bound Mixtures [81], which establishes the limits for shakedown (plastic shakedown limit) and plastic creep.

$$\epsilon_{p,5000}^{1}-\epsilon_{p,3000}^{1}=0.045\times 10^{-3}\;\mathrm{strain},\;\mathrm{define}\;\mathrm{the}\;\mathrm{plastic}\;\mathrm{shakedown}\;\mathrm{limit}$$

$$\epsilon_{p,5000}^{1}-\epsilon_{p,3000}^{1}=0.4\times 10^{-3}\;\mathrm{strain},\;\mathrm{define}\;\mathrm{the}\;\mathrm{plastic}\;\mathrm{creep}\;\mathrm{limit}$$

However, using these same reference values for tropical soils, particularly those with high clay content, may not be entirely appropriate. Therefore, in the present study, shakedown was considered to occur when the increment rate of permanent deformation reached approximately $10^{-7}$ m per load cycle, adopting a more conservative approach.

One of the main advantages of this methodology is its ability to classify soils based on their behavior concerning permanent deformation. The referenced studies categorize material behavior into three levels: A (shakedown), B (creep), and C (collapse).

As shown in the graph in Figure 13, test 9 initially exhibits behavior characteristic of type B during the early loading cycles, marked by well-defined plastic flow. However, as the number of load cycles increases, the increment rate of PD tends to stabilize, and the behavior transitions to type A, indicating a plastic shakedown process. Since the accumulated deformations in the initial cycles are relatively high, they cannot be disregarded, resulting in a mixed AB behavior, as suggested by Guimarães and Motta [21]. For the remaining samples, the observed behavior was predominantly type A, indicating plastic shakedown, with total permanent deformation remaining below 2.0 mm.

**Figure 14:**
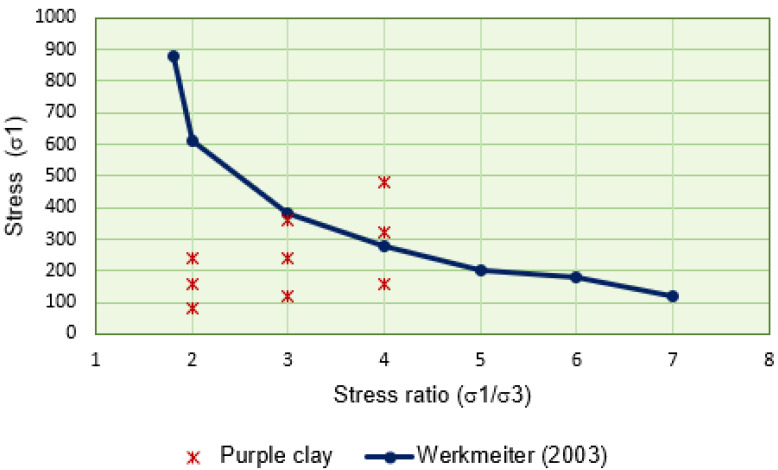
Comparison between the shakedown limit for the Granodiorite studied by WERKMEISTER [56] and the values obtained for Purple Clay.

Figure 14 presents a comparison between two datasets: (i) the shakedown limit obtained by Werkmeister et al. [80] and calculated according to the British standard BS 13286-7 [81], and (ii) the data points representing the nine tests conducted on Purple Clay in the present study. The shakedown limit is expressed in terms of the principal stress ratio $\sigma_{1}/\sigma_{3}$, where $\sigma_{1}=\sigma_{d}+\sigma_{3}$, as detailed in Table 1. Among the nine tests, only tests 6 and 9 resulted in stress ratios ($\sigma_{1}/\sigma_{3}$) equal to 4, placing them above the reference shakedown limit. Test 6 exhibited a clear shakedown condition, while test 9 indicated mixed (AB) behavior. These results suggest that the shakedown limit of Purple Clay is close to that of the reference granodiorite aggregate, further reinforcing the favorable performance of the studied material.

## 4. Conclusions

This study investigated the geotechnical properties of Purple Clay, a lateritic clay from the São Paulo region, Brazil, through detailed physical, chemical, and morphological characterization, as well as an evaluation of its mechanical behavior using repeated-load triaxial tests. The main conclusions are as follows:

- Mineralogical analyses revealed a significant presence of iron oxides, such as goethite and hematite, which contribute to the soil’s high density, mechanical strength, and cementation potential. These characteristics suggest that Purple Clay performs well in geotechnical applications, particularly in scenarios requiring long-term stability and resistance.
- The resilient modulus (RM) of Purple Clay compacted with energy equivalent to the standard Proctor test was found to be more influenced by confining stress than by deviatoric stress, with the best fit achieved using the combined model. Under these conditions, the average RM was 258 MPa. When compacted with energy equivalent to the intermediate Proctor test, the RM tended to stabilize, reaching an average value of 275 MPa.
- The RM of Purple Clay is of the same order of magnitude as some graded aggregates, based on values reported in the literature.
- The results of the permanent deformation (PD) tests indicate low accumulated PD, except under higher stress levels, where a hypothetical pavement layer composed of Purple Clay could contribute up to 35% of the total admissible rutting value.
- When compared to a graded aggregate from Chapecó, Purple Clay exhibited similar total permanent deformation levels under low-stress conditions but showed slightly higher deformation under higher stress states.
- The PD analysis results suggest that Purple Clay demonstrates good resistance to rutting under repeated loads, with total permanent deformations below 2.0 mm in most tests, indicating a tendency toward shakedown behavior. Although some tests displayed mixed (AB) behavior, Purple Clay’s performance compared favorably to the shakedown limit of a granodiorite-graded aggregate, demonstrating its suitability as a sub-base material.

Despite the promising results observed in this study, further investigations are necessary to fully assess the applicability of Purple Clay in pavement structures. A mechanistic-empirical analysis considering actual pavement design parameters, traffic loads, and climatic effects would provide a more comprehensive assessment of Purple Clay’s structural contribution and long-term performance in road applications.

## Figures and Tables

**Table 1:** List of PD tests conducted with Purple Clay.

Test	Stress (MPa)		Stress Ratio ($\sigma_{d}$/$\sigma_{3}$)	N
	$\sigma_{d}$	$\sigma_{3}$		
1	0.040	0.040	1	160.000
2	0.080		2	230.000
3	0.120		3	150.000
4	0.080	0.080	1	230.000
5	0.160		2	240.000
6	0.240		3	169.000
7	0.120	0.120	1	390.000
8	0.240		2	257.000
9	0.360		3	340.000

**Table 2:** Grain composition of Purple Clay.

Grain Composition (%)						
**Material**	**Clay**	**Silt**	**Sand**			**Stony**
			**Fine**	**Medium**	**Coarse**	
Purple Clay	37	25	29	8	1	0

**Table 3:** Physical–chemical analysis of the Purple Clay.

Sample	pH		%ΔP	Sulphuric Attack							
	**H_2_O**	**KCL** ** 1 M**		**SiO_2_** ** %**	**Al_2_O_3_** ** %**	**Fe_2_O_3_** ** %**	**TiO_2_** ** %**	**K_2_O** ** %**	**Res** ** %**	**Ki**	**Kr**
**Clay**	5.52	5.71	8.39	13	20.1	25.5	4.2	0.02	24.6	1.1	0.61

**Table 4:** The relationship between resilient modulus and MCT classification. Data from Neto et al. [74] compared with the results obtained in this study.

Soil	RM (MPa)	
	**Base (100% PI)**	**Subgrade (100% PN)**
LG’	100	90–160
LG’	200	110–220
LA’	220–300	160–220
LA’	220–300	-
LA’	270	170
LA	240	-
**Purple Clay from this study**	275	258

## Data Availability

The original contributions presented in this study are included in the article. Further inquiries can be directed to the corresponding author.
